# Compositional Discrimination of Decompression and Decomposition Gas Bubbles in Bycaught Seals and Dolphins

**DOI:** 10.1371/journal.pone.0083994

**Published:** 2013-12-19

**Authors:** Yara Bernaldo de Quirós, Jeffrey S. Seewald, Sean P. Sylva, Bill Greer, Misty Niemeyer, Andrea L. Bogomolni, Michael J. Moore

**Affiliations:** 1 Woods Hole Oceanographic Institution, Biology Department, Woods Hole, Massachusetts, United States of America; 2 Woods Hole Oceanographic Institution, Marine Chemistry and Geochemistry Department, Woods Hole, Massachusetts , United States of America; 3 Integrated Statistics, Woods Hole, Massachusetts, United States of America; 4 NOAA NMFS Northeast Fisheries, Woods Hole, Massachusetts, United States of America; 5 International Fund for Animal Welfare, Yarmouth Port, Massachusetts, United States of America; 6 University of Connecticut, Department of Pathobiology and Veterinary Science, Storrs, Connecticut, United States of America; Texas A&M University-Corpus Christi, United States of America

## Abstract

Gas bubbles in marine mammals entangled and drowned in gillnets have been previously described by computed tomography, gross examination and histopathology. The absence of bacteria or autolytic changes in the tissues of those animals suggested that the gas was produced peri- or post-mortem by a fast decompression, probably by quickly hauling animals entangled in the net at depth to the surface. Gas composition analysis and gas scoring are two new diagnostic tools available to distinguish gas embolisms from putrefaction gases. With this goal, these methods have been successfully applied to pathological studies of marine mammals. In this study, we characterized the flux and composition of the gas bubbles from bycaught marine mammals in anchored sink gillnets and bottom otter trawls. We compared these data with marine mammals stranded on Cape Cod, MA, USA. Fresh animals or with moderate decomposition (decomposition scores of 2 and 3) were prioritized. Results showed that bycaught animals presented with significantly higher gas scores than stranded animals. Gas composition analyses indicate that gas was formed by decompression, confirming the decompression hypothesis.

## Introduction

During the last decade, a wide range of evidence showing the presence of gas bubbles in marine mammals has challenged our understanding of their diving physiology [1-7]. Prior to these reports marine mammals were considered protected from decompression sickness due to anatomical, physiological, and behavioural adaptations.

Gas bubbles in marine mammals were first described in beaked whales stranded in temporal and spatial association with military exercises [1,3]. These authors described an acute and systemic gas and fat embolic syndrome similar to decompression sickness in human divers and proposed a behaviourally induced decompression sickness-like disease as a plausible causal mechanism. Acute and chronic gas bubble lesions were described in cetaceans single-stranded on the UK coast [2]. A decompression mechanism involving embolism of intestinal gas or *de novo* gas bubble formation from tissues supersaturated with N_2_ was proposed as a possible aetiology for these lesions. Dysbaric osteonecrosis, necrosis of a portion of the bone associated with exposure to large ambient pressure changes, was described in sperm whales [5]. Although the aetiology of dysbaric osteonecrosis remains unknown, it is generally accepted that gas embolism might be the prime cause [8]. Gas bubbles were later described in seals, dolphins and porpoises entangled and drowned at depth in gillnets [6]. Authors suggested that the gas was produced peri- or post-mortem by a fast decompression, probably by quickly hauling animals entangled in the net at depth to the surface. Dennison et al. (2012) found gas in kidneys and hepatic portal vasculature in live stranded dolphins using a B-mode ultrasound [7].

The interpretation of the gas bubble findings remains scientifically controversial. Alternatives to the hypothesis that marine mammals could suffer a decompression sickness-like disease include the entry of gas into the vascular system as a result of dissection, gas produced by Clostridium or other gas-producing bacteria, and putrefaction.

Investigations into the constituents of the gas bubbles as well as their amount and distribution could potentially clarify this controversy. Gas scoring and gas composition analysis are two new diagnostic tools for the study of gas embolism [9-11]. The gas score is an index-based method used to describe the gross amount of gas observed at necropsies [10,11]. This method discriminates between gases produced by decompression, air embolism, and putrefaction using an index to describe the amount of gas bubbles found intravascularly and extravascularly within the body at necropsies. Gas compositional analysis also discriminates different gas etiologies, but uses the composition and relative abundance of constituent gases to identify their source [9]. 

A method to study the gas composition of bubbles in bodies was recently adapted to marine mammal field necropsies [12]. By applying this method to beaked whales stranded in temporal and spatial association with military exercises and in splenic gas-chronic lesions from a *Grampus griseus* stranded in the UK coast, N_2_ was confirmed as the major constituent of the bubbles [12]. 

A study of gas scores in stranded marine mammals showed that bubbles are a common finding in necropsies of stranded cetaceans so that the quantity of the bubbles is more important than the mere presence of bubbles from a pathological point of view [11]. The gas score method was able to differentiate the cases diagnosed with “gas embolism” through complete pathological studies from other stranded cetacean cases with lower gas scores. 

In this study, we characterized the presence, amount, distribution and composition of the gas bubbles from bycaught marine mammals in anchored sink gillnets and bottom otter trawls. We compared these data with marine mammals stranded on Cape Cod to determine if gas bubbles observed in bycaught marine mammals come from supersaturated tissues or are produced *post-mortem* by putrefaction.

## Materials and Methods

The presence of gas bubbles and their composition was studied in bycaught marine mammals and compared to stranded marine mammals. Bycaught animals were received under NOAA Permit No. 932-1905/MA-009526. Stranded animals were received under authorization from NOAA Northeast Regional Office. The animals used in this study for research had already died before they become part of the project, therefore ethical approval was not required.

Bycaught marine mammals (n = 13) were obtained through the NOAA Northeast Fisheries Observer Program: short-beaked common dolphins (*Delphinus delphis*) (n = 7), harbour seals (*Phoca vitulina*) (n = 2), grey seals (*Halichoerus grypus*) (n = 2) and harbour porpoise (*Phocoena phocoena*) (n = 1). The dolphins were recovered from bottom otter trawls, while the other species were recovered from anchored sink gillnets. NOAA fisheries observers recorded depth, soak or tow duration, and water temperature (Table 1). On board, animals were placed on ice. Once the vessels returned to port, the animals were transported by road to Woods Hole, MA, USA, where necropsies were performed at different *post-mortem* times. For the study of stranded animals, the International Fund for Animal Welfare (IFAW) provided access to 27 marine mammals stranded along the coast of Cape Cod, MA, USA. Most of the stranded marine mammals were short beaked common dolphins (n=16). Stranded animals and their circumstances during the stranding that could potentially influence the formation of gas bubbles are reported in Table 2. 

**Table 1:** Identification number of the bycaught marine mammals included in the study, environmental data, and decomposition code.

**BYCAUGHT DATA**						
**ID number**	**Species**	**Gear Type**	**Depth (m)**	**Soak/Tow Duration (h)**	**Water Temp (°C)**	**Decomposition code**
D09069	*D. delphis*	Bottom Otter Trawl	64	3.5 h tow	9.4	2
D09385	*P. vitulina*	Anchored Sink Gillnet	42	24 h soak	8.8	2
D06585	*P. phocoena*	Anchored Sink Gillnet	77	72 h soak	5.0	2
D00193	*D. delphis*	Bottom Otter Trawl	29	2.6 h tow	5.0	3
D00195	*D. delphis*	Bottom Otter Trawl	29	2.6 h tow	5.0	3
D00196	*D. delphis*	Bottom Otter Trawl	29	2.6 h tow	5.0	3
D00197	*D. delphis*	Bottom Otter Trawl	29	2.6 h tow	5.0	2
D00198	*D. delphis*	Bottom Otter Trawl	29	2.6 h tow	5.0	2
D08031	*P. vitulina*	Anchored Sink Gillnet	29	96 h soak	5.5	2
D06200	*D. delphis*	Bottom Otter Trawl	31	2.8 h tow	11.6	2
D08334Dd	*D. delphis*	Bottom Otter Trawl	67.7	2.9 h tow	5.5	2
D09926Hg	*H. grypus*	Anchored Sink Gillnet	80.5	168.0 h soak	4.4	2
D09928Hg	*H. grypus*	Anchored Sink Gillnet	80.5	168.0 h soak	4.4	2

D = *Delphinus*, P = *Phoca* H = *Halichoerus.*

**Table 2:** Identification number of the stranded marine mammals included in the study, their stranding circumstances, and decomposition code.

**STRANDING DATA**					
**ID number**	**Species**	**Active stranding**	**Mass stranding**	**Re-stranded**	**Decomposition code**
IFAW12003Dd	*D. delphis*	No	yes	yes	2
IFAW12-007Dd	*D. delphis*	Yes	yes	no	3
IFAW12-033Dd	*D. delphis*	found dead	yes	yes	3
IFAW12-196Dd	*D. delphis*	found dead	yes	yes	3
IFAW12-198Dd	*D. delphis*	yes	yes	no	2
IFAW12-200Dd	*D. delphis*	yes	no	no	2
IFAW12-201Dd	*D. delphis*	found dead	no	yes	3
IFAW12-205Dd	*D. delphis*	yes	yes	no	2
IFAW12-206Pp	*P. phocoena*	yes	no	no	2
IFAW12-223Dd	*D. delphis*	yes	no	no	2
IFAW12-224Pp	*P. phocoena*	found dead	no	no	3
IFAW12-228Dd	*D. delphis*	no	yes	no	2
IFAW12-229Dd	*D. delphis*	yes	yes	no	2
IFAW12-273Pp	*P. phocoena*	yes	no	no	2
IFAW12-277Pv	*P. vitulina*	yes	no	no	2
IFAW12-278Pv	*P. vitulina*	yes	no	no	2
IFAW12-283Dd	*D. delphis*	yes	yes	yes	3
IFAW12-284Dd	*D. delphis*	yes	yes	yes	3
IFAW12-334Dd	*D. delphis*	yes	no	no	3
IFAW12-340Dd	*D. delphis*	yes	no	no	2
IFAW13-009Pv	*P. vitulina*	Found dead	no	no	2
IFAW13-012Dd	*D. delphis*	found dead	no	no	3
IFAW13-020Pp	*P. phocoena*	yes	no	no	2
IFAW13-041Gm	*G. melas*	yes	no	no	2
IFAW13-044La	*L. acutus*	yes	no	no	2
IFAW13-049Hg	*H. grypus*	found dead	no	no	2
IFAW13-052Dd	*D. delphis*	yes	no	no	2

D = *Delphinus*, P = *Phoca*, G = *Globicephala*, L = *Lagenorhynchus*, H = *Halichoerus*

### (a): Post-mortem examinations

The decomposition status of the animals was evaluated using a decomposition code from 1-5, where code 1 represents live animals (which becomes code 2 at death), code 2 represents animals that are extremely fresh (no bloating), code 3 represents animals with moderate decomposition (bloating, skin peeling but organs still intact), code 4 represents animals with advanced decomposition (major bloating, organs beyond recognition), and finally code 5 represents animals that no longer had organs present [13].

Dissection was undertaken following Bernaldo de Quirós et al. (2012) to limit severing of large veins and to allow for the description and sampling of the intra- and extravascular gas [14]. Severing of superficial veins, such as the superficial veins of the dorsal fin and flukes, does not necessarily introduce gas bubbles in the cardiovascular regions that we are studying. Although these superficial veins drain into the lumbo-caudal venous plexus that we sampled, the plexus showed an absence of gas bubbles in animals that have been dissected following the present dissection protocol [11,15].

The presence and amount of intravenous bubbles in the subcutaneous, mesenteric, and coronary veins and in the lumbo-caudal venous plexus was characterized with a score from 0-VI [10]. 

Score 0 describes an absence of gas bubbles within the vein. Score I describes the presence of an occasional small bubble found by carefully screening veins. Score II describes the presence of either very few bubbles, either small “discontinuities of blood” or both. The discontinuities are small sections of veins showing absence of red cells and associated haemoglobin but with clear liquid instead, presumably plasma from which the red cells have retracted. Veins usually present different degrees of collapse at these regions, which are absent of gas. Score III describes the presence of more abundant and larger discontinuities of blood. Score IV describes moderate presence of gas bubbles within a specific vein. Score V describes abundant presence of gas bubbles. Score VI describes complete sections of vessels filled with gas. 

Extravascular gas, usually presented as subcapsular gas, was scored from 0-3 [10], where “score 0 describes an absence of gas, score 1 describes scarce presence (affecting only one target organ), score 2 describes moderate presence of gas (affecting two or three organs), and score 3 describes abundant gas (affecting many different organs)”.

After gas scoring, gas was sampled following a standard protocol [10,14] with some adaptations to allow use of materials currently available in the USA. Gas within body cavities such as intestine or pterygoid sinuses was sampled by using a 2-mL additive free glass tube (Kendall Monoject^™^ blood collection tube, ref: 301116) with a BD vacutainer® single-use holder (ref: 364815) and a double pointed needle with a rubber barrier on the tube puncture side (BD vacutainer® eclipse™ blood collection needle, ref: 368607). Gas bubbles in veins were sampled with disposable insulin syringes (BD Plastipak U-100 insulin ref: 329651). Gas samples were transferred immediately into the additive free glass tubes. One new syringe and one new tube were used for each bubble. To sample gas from inside the heart chambers, an aspirometer (U201100896, custom made by Yankee Glassblower Inc., Concord, MA, USA) was used [12].

**Figure 1:**
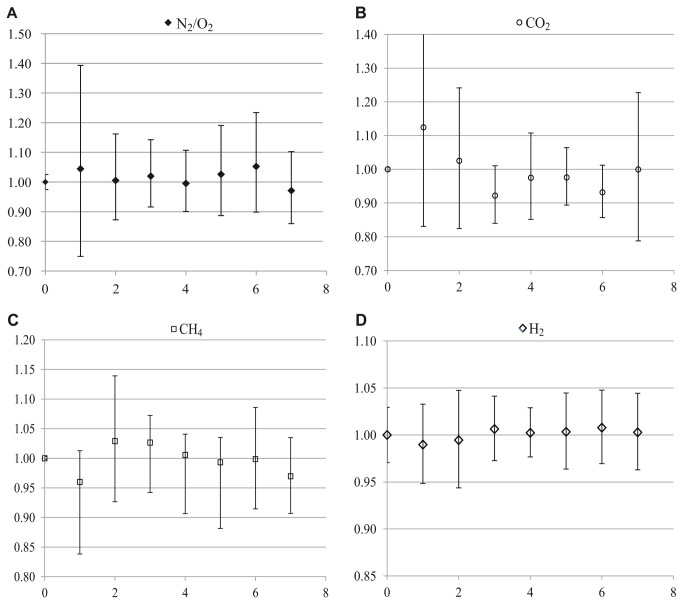
Preservation of different gases with time inside monoject tubes. Abundance of N_2_/O_2_ (a), CO_2_ (b), H_2_ (c), and CH_4_ (d) from the 2-mL additive free Kendall Monoject^™^ tube, normalized to the abundance of each gas at time zero.

Adaptations to materials currently available in the USA included the use of the 2-mL Monoject™ additive free glass tube instead of the 5-mL additive-free BD vacutainers® (ref: 367624) which are no longer available in the USA. The 2-mL Monoject™ were selected as a possible substitute of the 5-mL vacutainer® based on their similar features; both tube types are made of glass, additive free and of small volume. We tested the gas storage capability of the Monoject™ tubes by injecting 1ml of H_2_ 99.99% (Supelco, ref: 300100) and 0.5 ml of a SCOTTY specialty gas calibration standard containing 5% of CO_2_, CO, N_2_, and O_2_ and 4% of CH_4_, and H_2_ in a balance of helium (Supelco, ref: 501697), into 40 Monoject™ tubes. Tubes were stored at room temperature (22°C). Beginning on the day of injection, replicates were analyzed in sets of 5 each day. Ratios of gas levels (μmol) for each day relative to day zero were calculated to detect differences in gas composition over time (Figure 1). 

Bernaldo de Quirós et al. (2011) found near-significant differences in N_2_ content (P=0.056) between samples transported in the passenger cabin of a commercial jet aircraft and control samples, and recommended transporting samples inside a pressure resistant vessel to ensure that no changes in gas composition would occur during aircraft transportation [12]. To address this problem, we tested a potential transportation solution: a plastic housing resistant to negative pressures (PREVCO™ subsea housing). For this goal, 40 2-mL additive free tubes were filled with 1mL of air, with 20 of them being aircraft shipped (round trip). A barometer (High Gear Axio Black Altimeter Watch 20102HG) was transported with the samples in order to detect changes in pressure inside the plastic housing. The levels of O_2_ and N_2_ in both experimental conditions (airplane and control) were compared statistically. 

### (b): Gas analyses

Gas analyses were performed according to Bernaldo de Quirós et al. (2011), but with some modifications to the equipment available [12]. Two gas chromatographs (GC) were used to fully characterize gas composition. One GC, a Hewlett Packard 5890 Series II, was equipped with two packed columns. An 8’ x 1/8^”^ Hayesep Q 100/120 mesh was plumbed in parallel with a 12’ x 1/8” 5A molecular sieve 60/80 mesh connected. The gas sample was split following injection with CO_2_ and hydrocarbon compounds up to C4 (butane) separated on the Haysep Q-phase column, and permanent gases (O_2_, N_2_, Ar) separated on the 5A molecular sieve column. Permanent gases and CO_2_ were detected using a thermal-conductivity detector (TCD) while hydrocarbons were detected using a flame-ionization detector (FID) serially connected to the TCD. The temperature for the TCD was held at 250 °C, and for the FID at 300°C. The GC oven was held at an isothermal temperature of 40°C with helium, flowing at 30ml/min, as the carrier gas. Although this chromatograph can detect H_2_, the detection limit was not optimal. For this reason, a Shimadzu GC-8A equipped with 6’ x 1/8” 5A molecular sieve column, a TCD and N_2_ carrier gas was employed for H_2_ analysis. 

### (c): Statistical analyses

All data were analyzed using the PASW Statistics data analysis software, version 18.0.0. Hypothesis testing was considered significant at *P*<0.05. For comparison of two groups of small sample size the U-Mann Whitney Rank Sum Test was applied. For linear correlation studies the Spearman’s test was chosen. Two tails were considered for all statistical analyses.

## Results

### (a): Methodology innovations

The 2-mL additive free Monoject™ tube was explored as an alternative to the 5-mL additive-free BD vacutainer®. No differences in gas content (μmol) were found throughout the 7 days of the study as indicated by the lack of variation in gas abundances normalized to the time zero values (Figure 1). 

A plastic housing resistant to negative pressures was tested for its suitability for transportation of gas samples in an aircraft. No significant differences were found for either O_2_ (P= 0.385) or N_2_ (P= 0.915) in control samples compared to aircraft shipped samples in the pressure vessel. The barometer registered a maximum pressure difference of a simulated altitude of 30 m.

### (b): Prevalence of bubbles

Gas bubbles were observed in 85% (34/40) of the examined marine mammals. All bycaught marine mammals presented with gas bubbles (13/13), and 77.8% (21/27) of the stranded marine mammals presented with gas bubbles. The highest prevalence of gas bubbles, within the different anatomical regions studied (subcutaneous, mesenteric, coronary veins, veins of the lumbo-caudal plexus, and subcapsularly in the organs), was found subcapsularly in the peri-renal region in 80% of the animals (32/40). Absence of peri-renal subcapsular emphysema was observed in two bycaught and in six stranded marine mammals. There was no apparent relationship between the prevalence of peri-renal subcapsular gas and the prevalence of gas bubbles in the lumbo-caudal venous plexus. An absence of gas bubbles in the lumbo-caudal venous plexus was observed in eight animals in which peri-renal subcapsular gas was present. In contrast, gas bubbles were observed in the lumbo caudal venous plexus in four animals in which peri-renal subcapsular gas was absent. The second highest prevalence of gas bubbles was found in the mesenteric veins, and the lowest in the coronary veins. There were no differences in the distribution pattern of the gas bubbles between stranded and bycaught marine mammals within the studied anatomical regions. 

### (c): Gas scores

**Figure 2:**
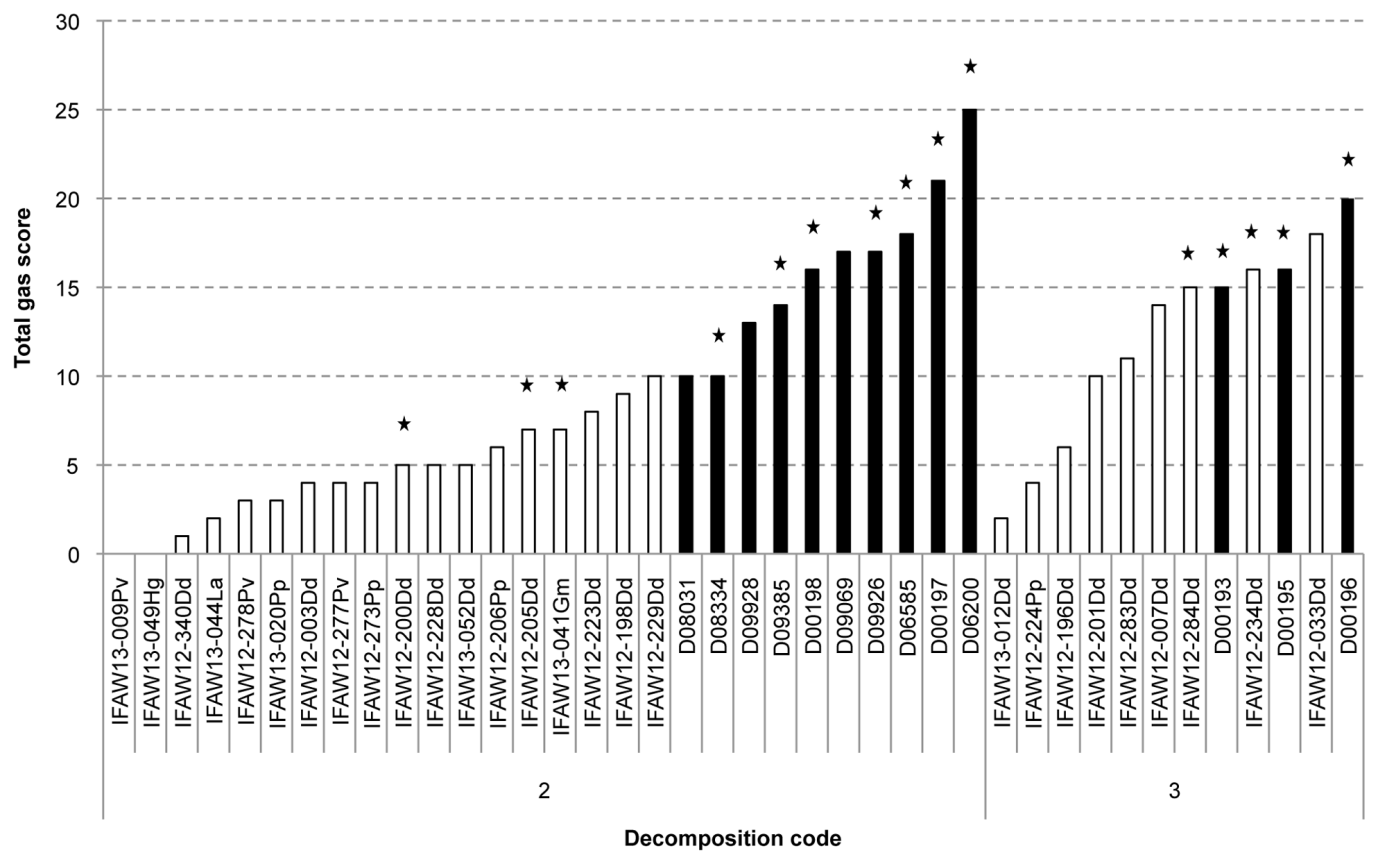
Amount of gas in stranded and bycaught marine mammals. Total gas score (y axis) of stranded (white bars) and bycaught (black bars) marine mammals according to decomposition code. The stars represent those animals from which gas samples were taken and analyzed.

Bycaught marine mammals presented with higher gas scores than stranded marine mammals (Figure 2). Differences between decomposition code 2 bycaught and stranded marine mammals were statistically significant (P <0.001). The highest gas score for stranded marine mammals with decomposition code 2 was 10, with a median value of 4. In contrast, 10 was the lowest recorded gas score for a bycaught animal with decomposition code 2. Gas scores for bycaught marine mammals ranged between 10-25, with a median value of 16. Differences between groups decreased with decomposition code. No significant differences in gas score were found between stranded and bycaught animals with decomposition code 3 (P=0.078). Median values for stranded and bycaught marine mammals with decomposition code 3 were 11 and 16, respectively. 

All bycaught marine mammals from this study presented with gas bubbles. There was no correlation between gas score and depth of the net (ρ=-0.176; P=0.626) or temperature of the water (ρ=0.121; P=0.739). The distribution of the gas score was the same for animals trapped in gillnets and trawls (P=0.422).

Higher volumes of gas were recovered from the right cardiac ventricle compared to the left, by using an aspirometer [12]. In the freshest case (D06200), 20 mL of gas were found in the right cardiac ventricle compared to 3 mL in the left cardiac ventricle.

### (d): Gas composition of bubbles

Gas samples were collected from 9 bycaught and 5 stranded marine mammals. They were all short beaked common dolphins except for one harbour porpoise, one long-finned pilot whale and two grey seals. Nine of the animals presented with a decomposition code of 2, the other five presented with a decomposition code of 3. Specimens from which gas samples were collected are indicated with a star above their gas score bar in Figure 2.

The gas composition of bubbles sampled from bycaught and stranded marine mammals were very similar. Given that bycaught animals are the focus of this study, we will describe those in detail. From the ten studied bycaught marine mammals, four of them were very fresh animals (D06585, D06200, D09928 and D09926), while two other animals (D00197 and D00198) were less fresh, but still considered code 2. A decomposition code 3 was given to D00196 and D00195. D00193 was slightly more decomposed than D00196 and D00195.

**Figure 3:**
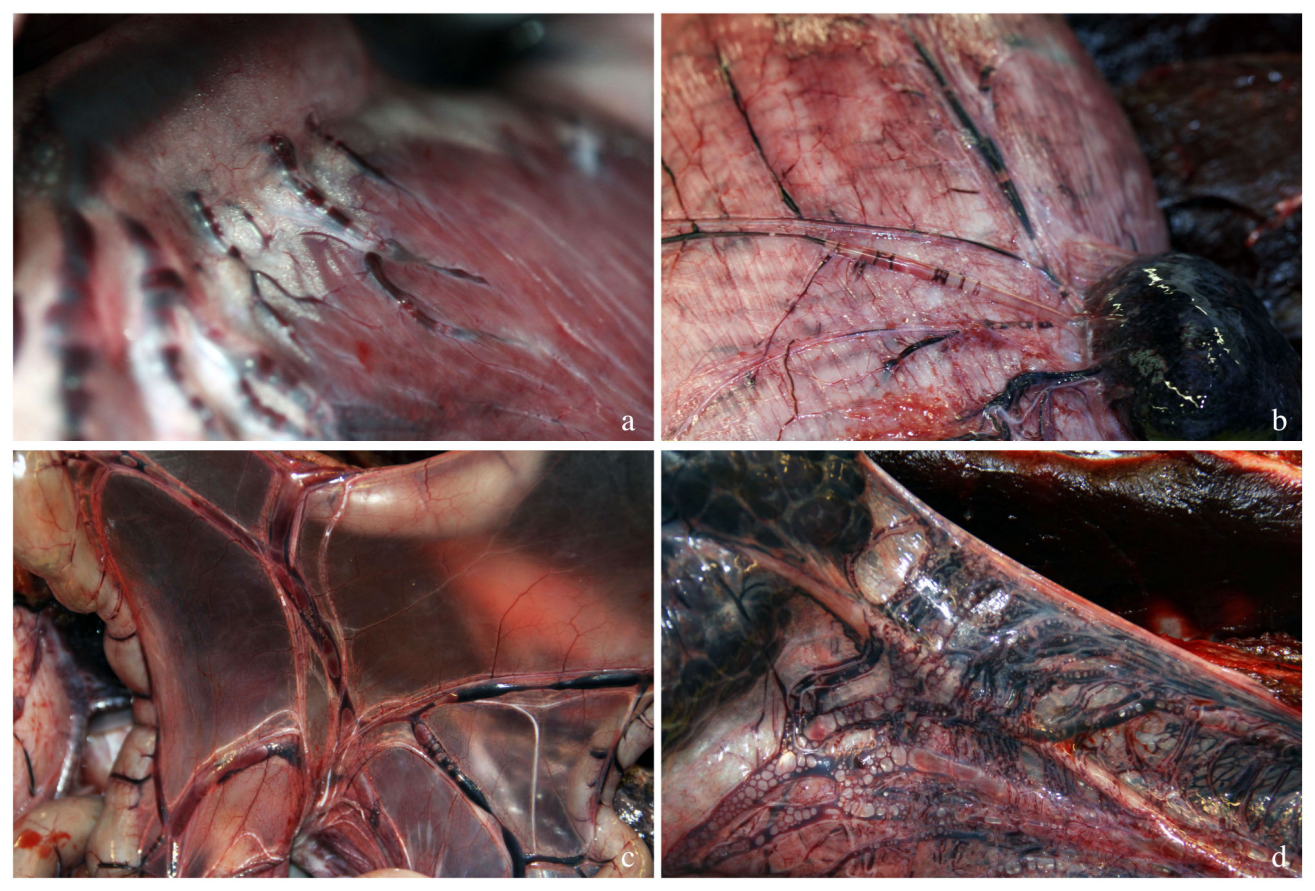
Photographs of gas bubbles in different tissues. Gas bubbles in the coronary (a), gastric (b), mesenteric veins(c), and in the lumbo-caudal venous plexus (d) of D06200, a bycaught short beaked common dolphin.

**Figure 4:**
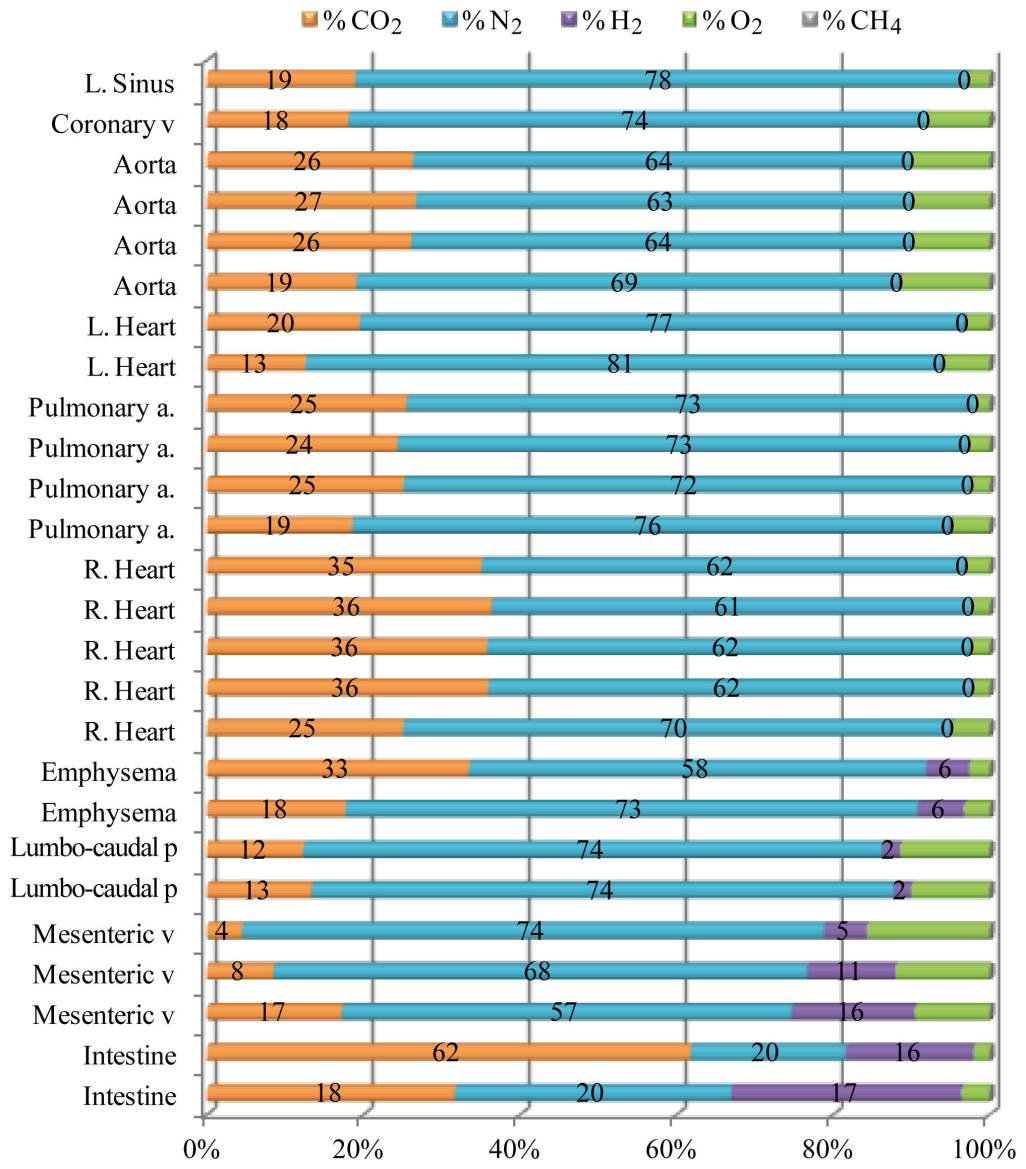
Gas composition of the bubbles found in D06200. Relative gas composition (% μmol) of samples taken at different body locations of D06200, a bycaught short beaked common dolphin. Necropsy was performed within 12 h post mortem. Abbreviations: L, left; R, right; v, vein; a, artery; p, plexus.

D06200 was a very fresh animal. This dolphin was recovered on board within 2.8 hours of death and the necropsy commenced within 12 hours post-mortem. This dolphin presented with the highest gas score from this study (Figure 2). Gas bubbles were widely dispersed through all the veins enabling the sampling of gas bubbles from different intra- and extra-vascular locations (Figure 3). This animal illustrates how the composition of the constituents varies according to location (Figure 4). Other fresh animals (D06585, D09928, and D09926) presented a similar gas composition as D06200 (Figures S1-3).

Most of the samples were composed of N_2_, CO_2_ and some O_2_. CH_4_ was absent, or present in quantities lower than 0.1% mol. H_2_ was only present in the intestine, mesenteric veins, lumbo-caudal venous plexus, and in the peri-renal emphysema. N_2_ was the main compound in all the gas samples except for the intestinal gases where CO_2_ was the main constituent. The lowest levels of N_2_ were found in the mesenteric veins and in the peri-renal emphysema (57-58 mol %), followed by the samples recovered from the right cardiac ventricle (61 mol %). The highest N_2_ levels were found in the left cardiac ventricle (77-81 mol %) and in the pterygoid sinus (78 mol %). In general, N_2_ mol % ranged between 60-80. In samples where H_2_ was present, N_2_ levels were still high (57-74 mol %). Samples from the right cardiac ventricle had the highest CO_2_ content.

D00198 (Figure S4), D00197 (Figure S5), D00196 (Figure S6), D00195 (Figure S7), and D00193 (Figure S8) are all short beaked common dolphins that were bycaught on the same bottom otter trawl trip. Necropsies of these animals were scheduled at increasing *post-mortem* times to assess patterns in the production and dissemination of putrefaction gases within the body.

**Figure 5:**
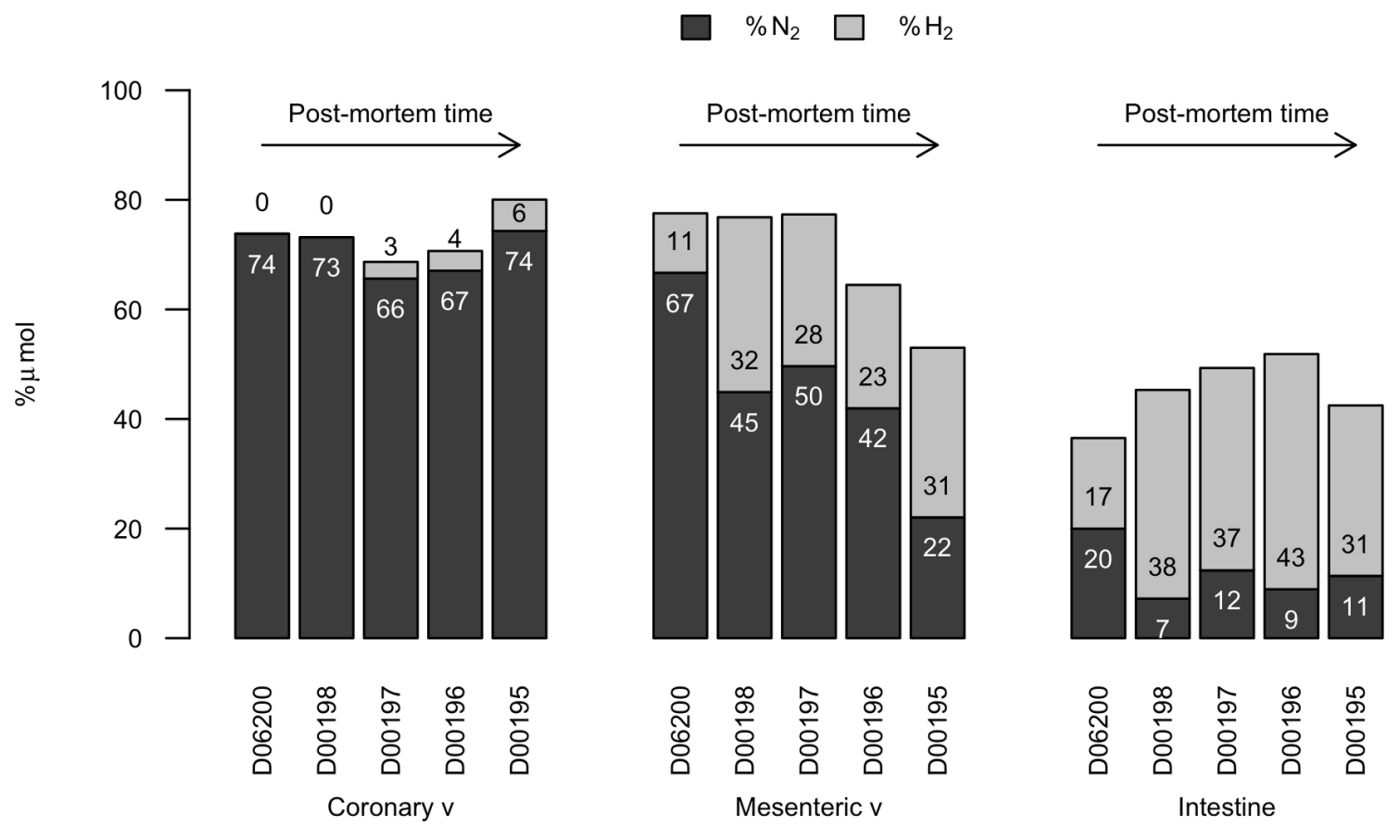
Relative composition of N_2_ and H_2_ in gas bubbles. N_2_ and H_2_ concentration in the coronary, mesenteric veins and in the intestine lumen of five animals with decreasing conservation status.

 When exploring gas composition in all bycaught animals with increasing putrefaction codes, we could observe how masking by putrefaction gases occurred first in the mesenteric veins (Figure 5). The gas composition of the bubbles found in the mesenteric veins of animals with a decomposition code of 2-3 had approximately 25 mol % H_2_ and 45-50 mol % N_2_. In animals with decomposition code 3, N_2_ levels in the mesenteric veins decreased further while H_2_ kept increasing. D00195 had N_2_ average levels of 22 mol % and 31 mol % of H_2_. The gas composition of the bubbles in the mesenteric veins became more similar to the composition of the intestinal gases with increased decomposition code. In contrast, the gas composition of the bubbles found in the vasculature of the thoracic cavity was less affected by putrefaction gases with the same decomposition codes H_2_ content increased slightly with decomposition code but N_2_ levels were still high (higher than 65 mol %) for decomposition code 3 animals (Figure 5).

## Discussion

This study has shown that bycaught marine mammals undergo a gas embolization process following decompression of supersaturated tissues marked by high nitrogen and carbon dioxide concentrations of the observed gas bubbles. Where decomposition is significant this process is overlain by a putrefactive increase in hydrogen content, which is most marked in the regions adjacent to the intestines. Issues to discuss in this regard include modifications to previous protocols, bubble prevalence and composition, and lily reasons for these observations.

### (a): Methodology

The 2-mL additive free Monoject™ tubes were shown to be as valid for gas storage as the 5-mL additive-free BD vacutainer® recommended by Bernaldo de Quirós et al. (2011) [12]. The use of the plastic housing protects the tubes against pressure changes, allowing cargo aircraft transportation. This methodological development will allow researchers worldwide to collect gas bubbles and to ship them to external laboratories for analyses within one week since collection. 

### (b): Prevalence of bubbles

The prevalence of bubbles in this study was very high (85% of stranded mammals). For stranded marine mammals 77.8% presented with gas bubbles, a value that is higher than the 58% reported by Bernaldo de Quirós et al. (2012) [11]. Furthermore, in the latter study authors reported a higher prevalence of gas bubbles in deep diving compared to non-deep diving marine mammals. The animals included in our study are non-deep divers, thus the difference in prevalence between both studies is remarkable. This difference is most likely attributed to the methodological approach; the study from 2012 was done retrospectively using photos, while the present study was performed prospectively searching for the bubbles during the necropsy. Bernaldo de Quirós et al. (2012) suggested that it is not uncommon to find intravascular +698*/bubbles in fresh necropsied cetaceans in contrast to fresh necropsied domestic animals or humans [16,17], and that the amount of intravascular gas bubbles in stranded cetaceans is more important than the mere presence of gas bubbles from a pathological perspective [11]. Our results reinforce this observation since 77.8% of the stranded marine mammals presented with gas bubbles, but none of them had gas bubbles in large amounts. In contrast, all bycaught marine mammals presented with large amounts of gas bubbles corresponding to high gas scores. The difference in gas score between fresh bycaught marine mammals compared to fresh stranded marine mammals was significantly higher (P< 0.001). Therefore this gas needs to be related to the bycatch itself and not to the putrefaction process. This result is in agreement with Cole et al. (2006), who did a series of experiments studying the post-mortem formation of bubbles in animals killed after compression exposure, but prior to decompression [18]. The authors could appreciate the presence of gas bubbles only in the subjected group at 1 and 8 h post-mortem. At 24 h post-mortem gas bubbles were also present in the control group but they were in larger quantities in the subject group. 

An alternative explanation would be a barotrauma (gas expanding from the lungs and entering the arterial circulation). We consider this hypothesis unlikely, since marine mammals are free divers, which take a breath at the surface (1 atm), and then they dive. They are not breathing compressed air from a tank at depth that will expand beyond lung volume with decreasing pressures. Since in breath hold divers initial and ending pressure is 1 atm, the physical requirements for overexpansion of the gas in the lungs are not fulfilled. Cowan and Walker (1979) proposed that alveolar rupture due to violent respiratory movement associated with asphyxiation could occur in dolphins trapped in nets, enabling the entry of gas into the blood-circulation [19]. However, during arterial gas embolisms caused by barotraumas, most of the gas is observed in the arterial side of the circulation instead of the venous side [20]. The gross visual evaluation of gas bubbles does not allow for assessment of presence of gas bubbles in the arterial side due to the opacity of arterial walls. However, we could determine the volume of gas found in the cardiac ventricles by using an aspirometer. We found much higher volumes of gas in the right cardiac ventricle compared to the left, suggesting that the source of the gas is the tissues. We could also observe gas bubbles in the peripheral veins. This observation is frequent in decompression cases [10,21-23] in contrast to air-embolism cases [10]. However, we do not rule out that some of the gas observed in the arterial side of the circulation could have come from the lungs.

Moore et al. (2009) proposed that the presence of gas bubbles in bycaught marine mammals was peri- or post mortem phenomenon produced by a fast decompression, probably by hauling animals entangled in the net [6]. No correlation between depth or water temperature and gas score was found. The shallowest depth from our study was 31 m, and the deepest was 77 m. Similar results have been found in experimental animal models with similar conditions to bycatch, even at shallower depths. Pigs and sheep compressed to a simulated depth of 18 m, allowing for a bottom time of at least 30 minutes, killed, and later decompressed, showed extensive bubble formation [18,24]. In contrast, gas bubbles were not observed in pigs with a bottom time of 10 minutes at a simulated depth of 18 m, even if the animals remained under pressure for 50 more minutes after their death [24]. An absence of gas bubbles were reported in pigs and sheep killed at atmospheric pressure, compressed to a simulated depth of 18 m, and decompressed [18,24]. These findings led Brown et al. (1978) to conclude that exposure to increased pressure for a minimum time before death is required for bubbles to form [24]. 

Brown et al. (1978) concluded from their results that ascent rates during recovery of animal bodies does not significantly influence bubble formation [24]. Our results support this conclusion since all bycaught marine mammals presented with gas bubbles regardless of their soak or duration time. If the animals died at depth, the rate of ascent is not expected to be of importance given that there is no blood circulation or pulmonary ventilation to wash out N_2_. Thus, a small change in pressure will result in the formation or expansion of gas bubbles. The only requirement for this to take place would be supersaturation of the tissues. However, if the animal is alive while the net is hauled, the rate of ascent might be of greater importance. 

### (c): Gas composition of bubbles

The gas composition of bubbles in the freshest animals confirmed that the main constituent was N_2_, indicating that the bubbles were formed by the off-gassing of saturated tissues. The gas composition results also showed how putrefaction gases evolve through the organism, demonstrating that thoracic veins, and specially the coronary veins, are the last to be affected by putrefaction overlying and masking the decompression gas signature.

The gas composition of the bubbles of the freshest animals showed that N_2_ was the main compound (representing up to 60-80% mol of the sample) followed by CO_2_ and some O_2_. These results are consistent with both, air embolism and gases from decompression [9,11]. Gas composition alone cannot discriminate between these two processes [9,25-33]. However, based on the highest presence of volume of gas found in the right cardiac ventricle compared to the left, and the involvement of peripheral veins, the most likely source of the bubbles was decompression of saturated tissues as previously discussed. 

The study of the gas composition of bubbles sampled from different locations within the same animal showed how putrefaction gases evolved in an organism. Putrefaction gases are mainly produced by enterobacteria, which are fermentative H_2_-producing bacteria that spread from the intestines throughout the rest of the body. Although a clear understanding of post-mortem bacterial movement remains elusive, it is thought to occur primarily via the blood and lymphatic vessels [34,35]. In a post mortem bacteria translocation study cultures were taken from the intestines, mesenteric lymph nodes, spleen, liver, kidney and cardiac blood at different *post-mortem* times [35]. The highest rates of culture-positive samples in the conventional mice were found in the mesenteric lymph nodes (59/80) followed by the kidney (35/80), liver (32/80), spleen (30/80) and cardiac blood (25/80). However on “humanized” mice, gnotobiotic mice to which a complex human microbiota was provided with, highest culture-positive rates were found in mesenteric lymph nodes (75/82) followed by the liver (50/82), cardiac blood (40/82), kidney (36/82), and spleen (34/82). These authors concluded that the time course and frequency of bacterial translocation into extra-intestinal organs is mainly determined by *post-mortem* changes in the GI tract which in turn affects the composition of the intraluminal microbiota composition [35]. Furthermore, different bacteria have different invasive properties; *Clostridium perfringens* has a doubling time of 8 minutes compared to the 20 standard minutes for other bacteria [36]. 

 In our gas samples, the highest rate of hydrogen detection was always found in the mesenteric veins (33/33 gas samples with a composition of 25.4 ± 11.7 %) compared to other locations within the body, except for the intestinal lumen. The mesenteric veins were also the cardiovascular location with highest prevalence of intravenous bubbles. This suggests bacterial translocation from the intestines through the mesenteric veins. Limitations of the method (presence of gas bubbles in the desired locations and volume of the gas bubbles) do not allow us to explore all the vascular regions in the different animals, therefore we have to study bacterial translocation in a case-by-case basis. For this purpose, case D06200 would be the best option since it was the freshest animal and with a very high abundance of gas bubbles (measured by gas score) enabling gas sampling from different locations. The primary vascular location with hydrogen was the caudal-venous plexus. Hydrogen was also present in the peri-renal emphysema. In contrast no hydrogen was found in either the large vessels of the thoracic cavity or the heart and coronary veins.

 In this animal, which was very fresh (decomposition code 2, with necropsy performed in less than 12 h *post-mortem*), the content of N_2_ at the mesenteric and renal veins was high (60-80% mol) regardless of the presence of H_2_. In contrast, in less fresh animals (decomposition code 2 but not as fresh as 12 h post-mortem), the presence of higher levels of H_2_ affected the N_2_ abundance at these locations. In animals with decomposition code 3 the relative % of N_2_ decreased (<45 mol %) due to the increase in H_2_ and CO_2_ levels. However, the coronary veins were one of the last locations affected by putrefaction gases. H_2_ was absent in the freshest cases (decomposition code 2) and present at very low concentrations in animals with incipient putrefaction (decomposition code 3) (Figure 4). As a result, high N_2_ levels (60-80 mol %) could be detected in the coronary veins of these animals. This result is of high relevance, since it has direct implications for sampling methodology. Gas sampling of coronary veins should be prioritized *versus* other locations for diagnostic purposes in order to avoid putrefaction-masking gases. Diagnostic attempts could be made in decomposition code 3 cases if gas samples are taken from the coronary or thoracic veins.

In summary, the amount of bubbles in bycaught marine mammals was significantly higher than that of stranded marine mammals. The combination of gas composition and distribution within the freshest animals suggested that these bubbles were formed from supersaturated tissues. Putrefaction gases appeared first in the mesenteric veins and last in the coronary veins, indicating that the bubbles in the coronary veins are of higher diagnostic value, especially in animals with a moderate extent of decomposition. 

## Supporting Information

Figure S1 **Gas composition of the bubbles found in D06585.** Relative gas composition (% μmol) of samples taken at different body locations of D06585, a bycaught short beaked common dolphin. Abbreviations: v, vein.
(TIF)

Figure S2 **Gas composition of the bubbles found in D09928.** Relative gas composition (% μmol) of samples taken at different body locations of D09928, a bycaught grey seal. Abbreviations: L, left.
(TIF)

Figure S3 **Gas composition of the bubbles found in D09926.** Relative gas composition (% μmol) of samples taken at different body locations of D09926, a bycaught grey seal. Abbreviations: L, left; v, vein.
(TIF)

Figure S4 **Gas composition of the bubbles found in D00198.** Relative gas composition (% μmol) of samples taken at different body locations of D00198, a bycaught short beaked common dolphin. Abbreviations: L, left; v, vein.
(TIF)

Figure S5 **Gas composition of the bubbles found in D00197.** Relative gas composition (% μmol) of samples taken at different body locations of D00197, a bycaught short beaked common dolphin. Abbreviations: v, vein.
(TIF)

Figure S6 **Gas composition of the bubbles found in D00196.** Relative gas composition (% μmol) of samples taken at different body locations of D00196, a bycaught short beaked common dolphin. Abbreviations: v, vein.
(TIF)

Figure S7 **Gas composition of the bubbles found in D00195.** Relative gas composition (% μmol) of samples taken at different body locations of D00195, a bycaught short beaked common dolphin. Abbreviations: v, vein.
(TIF)

Figure S8 **Gas composition of the bubbles found in D00193.** Relative gas composition (% μmol) of samples taken at different body locations of D00193, a bycaught short beaked common dolphin. Abbreviations: v, vein.
(TIF)
