# Supplementary figures and images for: Compositional Discrimination of Decompression and Decomposition Gas Bubbles in Bycaught Seals and Dolphins
Source: PLoS One. 2013 Dec 19;8(12):e83994. doi: 10.1371/journal.pone.0083994 (PMC3868626; doi:10.1371/journal.pone.0083994)

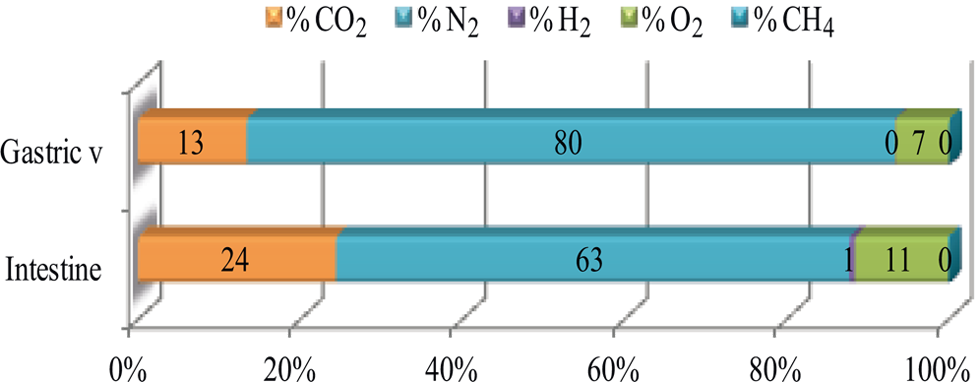

Supplement: Figure S1 — Gas composition of the bubbles found in D06585. Relative gas composition (% μmol) of samples taken at different body locations of D06585, a bycaught short beaked common dolphin. Abbreviations: v, vein. (TIF) [file pone.0083994.s001.tif]

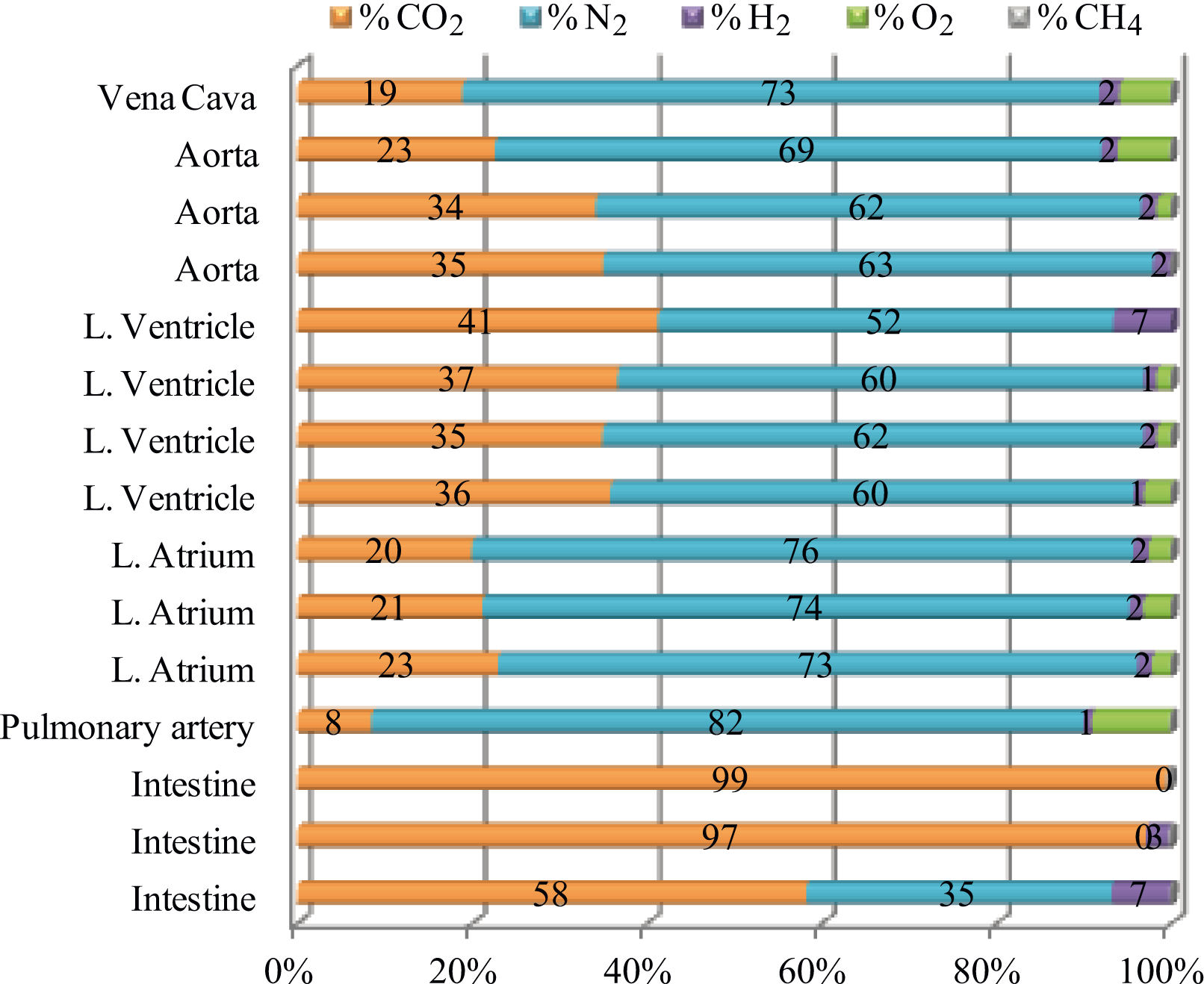

Supplement: Figure S2 — Gas composition of the bubbles found in D09928. Relative gas composition (% μmol) of samples taken at different body locations of D09928, a bycaught grey seal. Abbreviations: L, left. (TIF) [file pone.0083994.s002.tif]

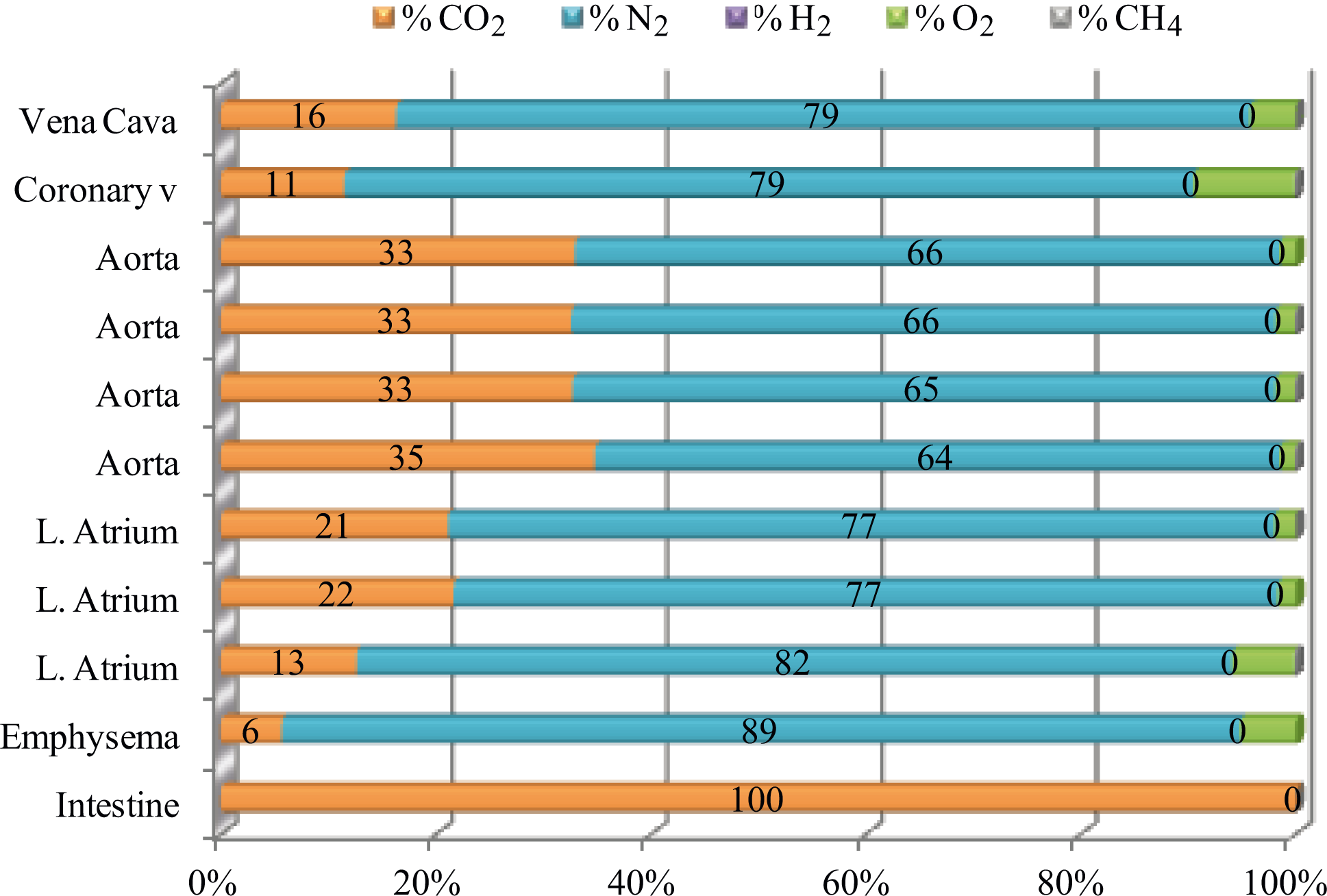

Supplement: Figure S3 — Gas composition of the bubbles found in D09926. Relative gas composition (% μmol) of samples taken at different body locations of D09926, a bycaught grey seal. Abbreviations: L, left; v, vein. (TIF) [file pone.0083994.s003.tif]

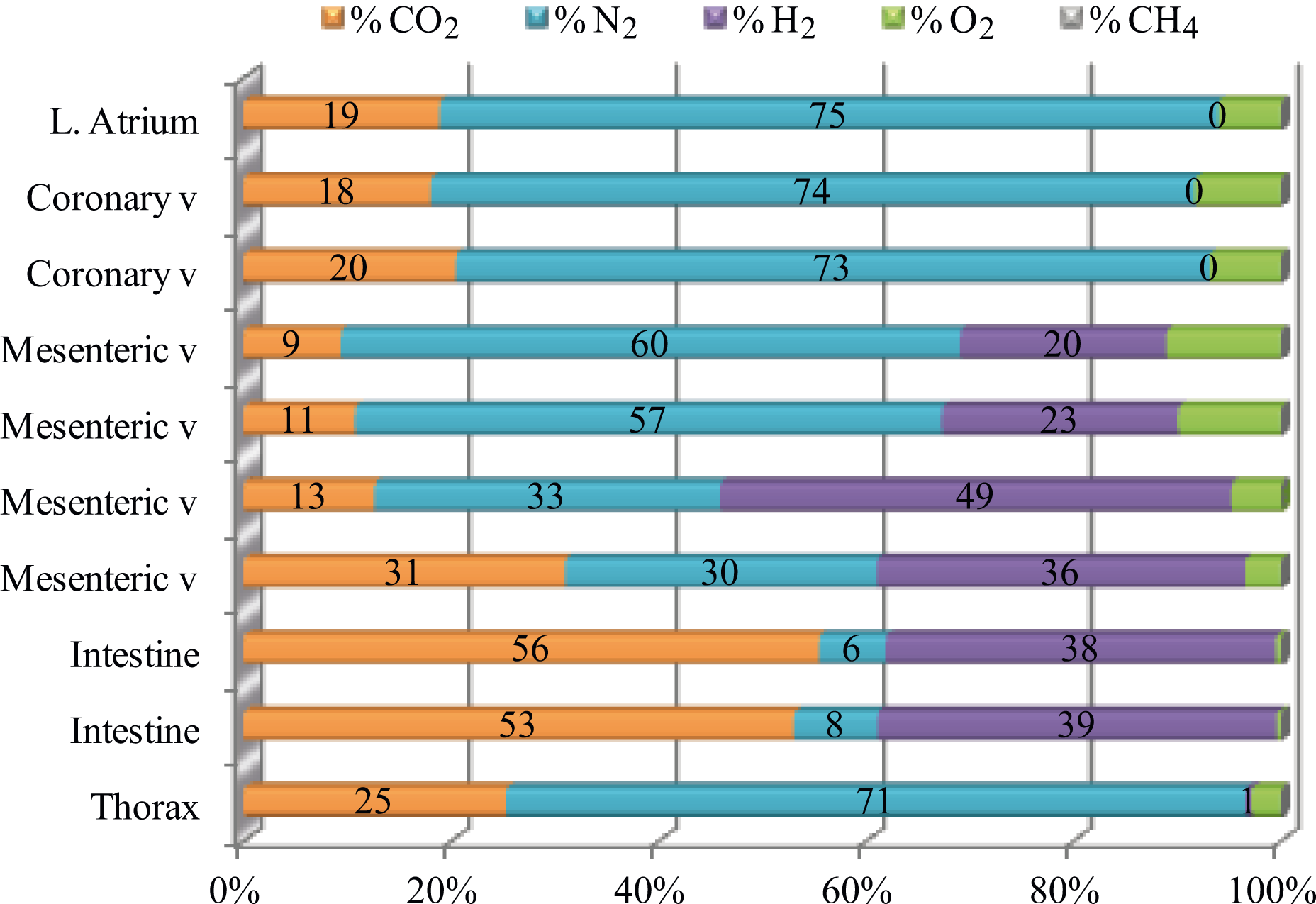

Supplement: Figure S4 — Gas composition of the bubbles found in D00198. Relative gas composition (% μmol) of samples taken at different body locations of D00198, a bycaught short beaked common dolphin. Abbreviations: L, left; v, vein. (TIF) [file pone.0083994.s004.tif]

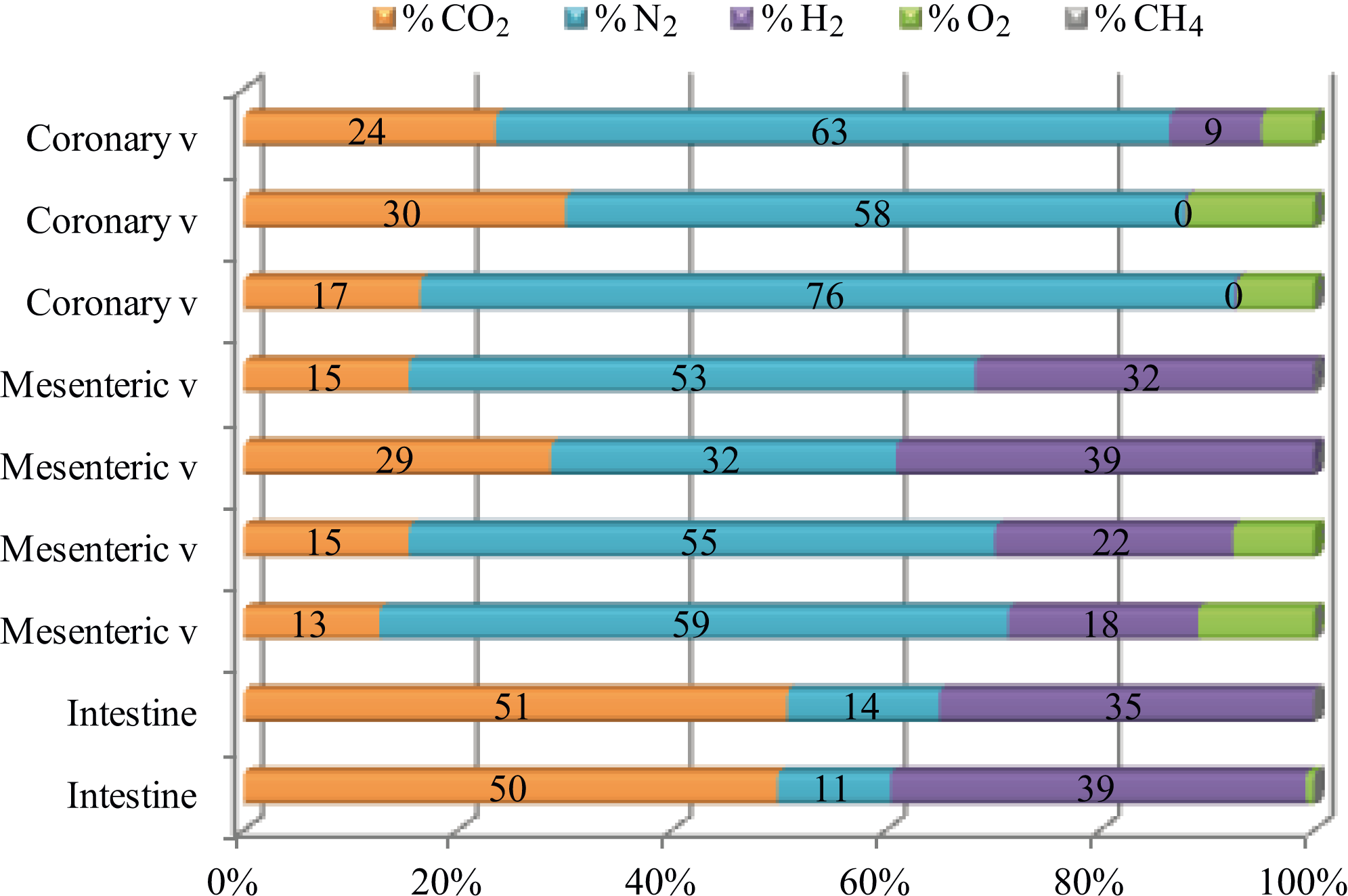

Supplement: Figure S5 — Gas composition of the bubbles found in D00197. Relative gas composition (% μmol) of samples taken at different body locations of D00197, a bycaught short beaked common dolphin. Abbreviations: v, vein. (TIF) [file pone.0083994.s005.tif]

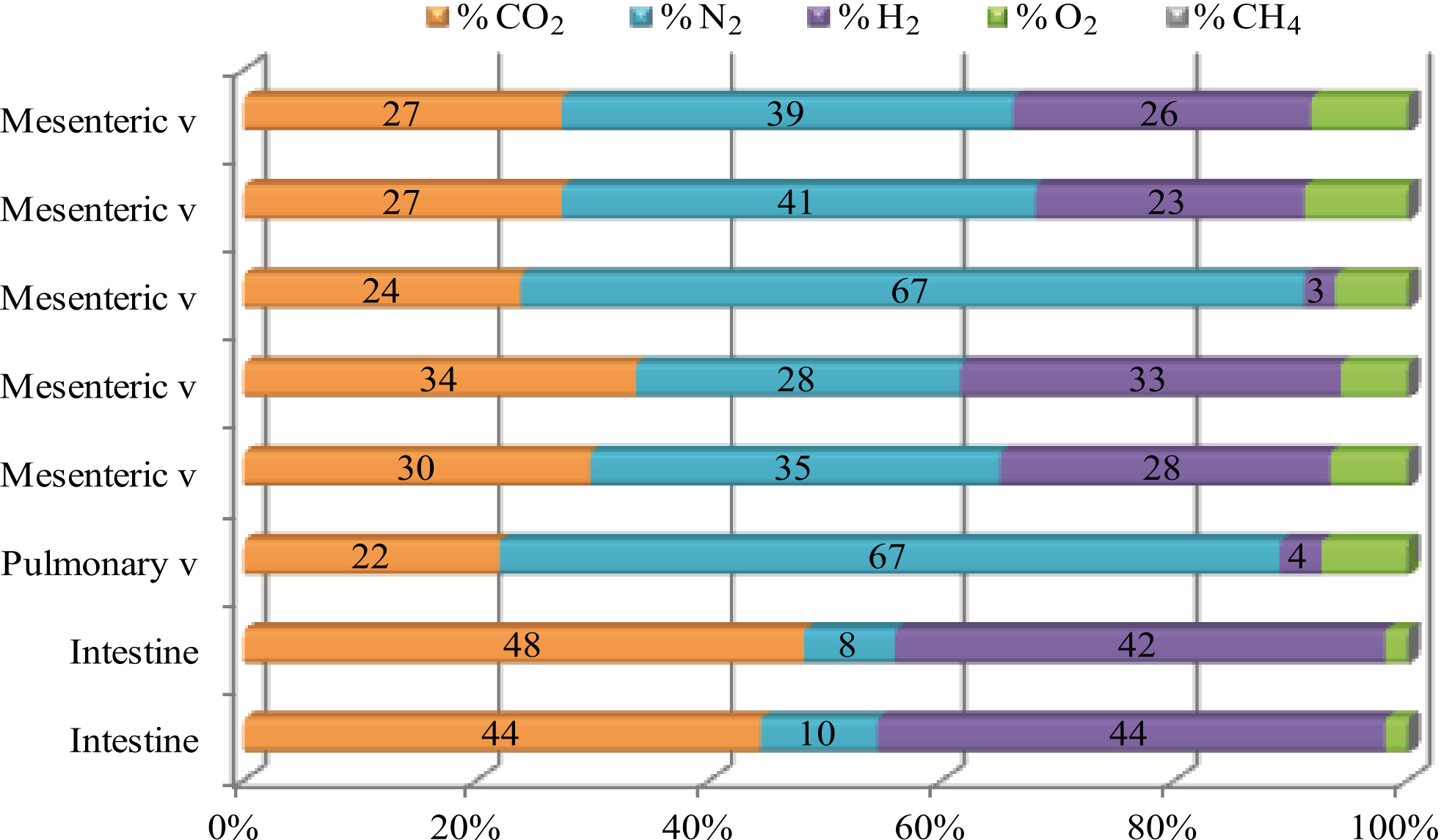

Supplement: Figure S6 — Gas composition of the bubbles found in D00196. Relative gas composition (% μmol) of samples taken at different body locations of D00196, a bycaught short beaked common dolphin. Abbreviations: v, vein. (TIF) [file pone.0083994.s006.tif]

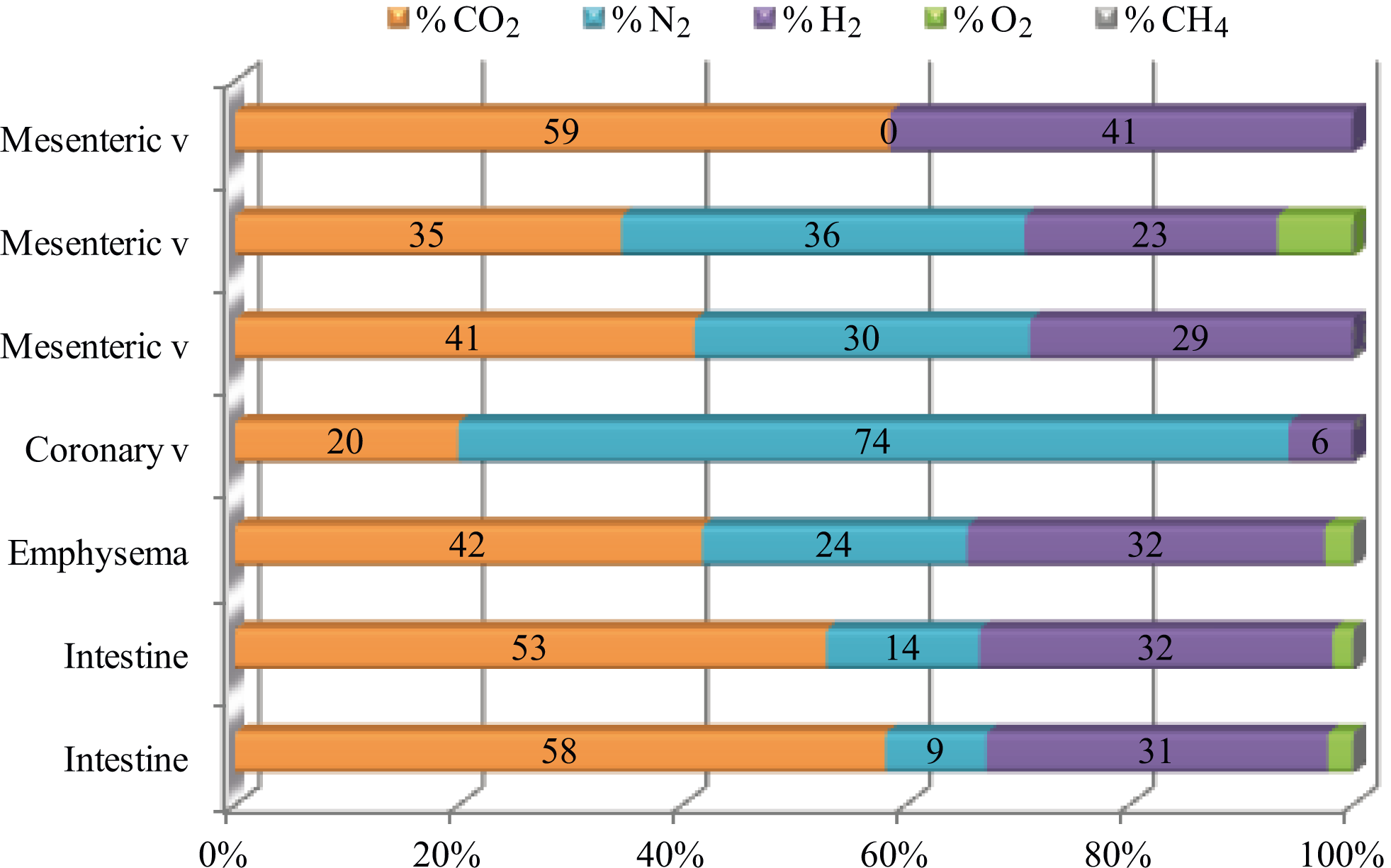

Supplement: Figure S7 — Gas composition of the bubbles found in D00195. Relative gas composition (% μmol) of samples taken at different body locations of D00195, a bycaught short beaked common dolphin. Abbreviations: v, vein. (TIF) [file pone.0083994.s007.tif]

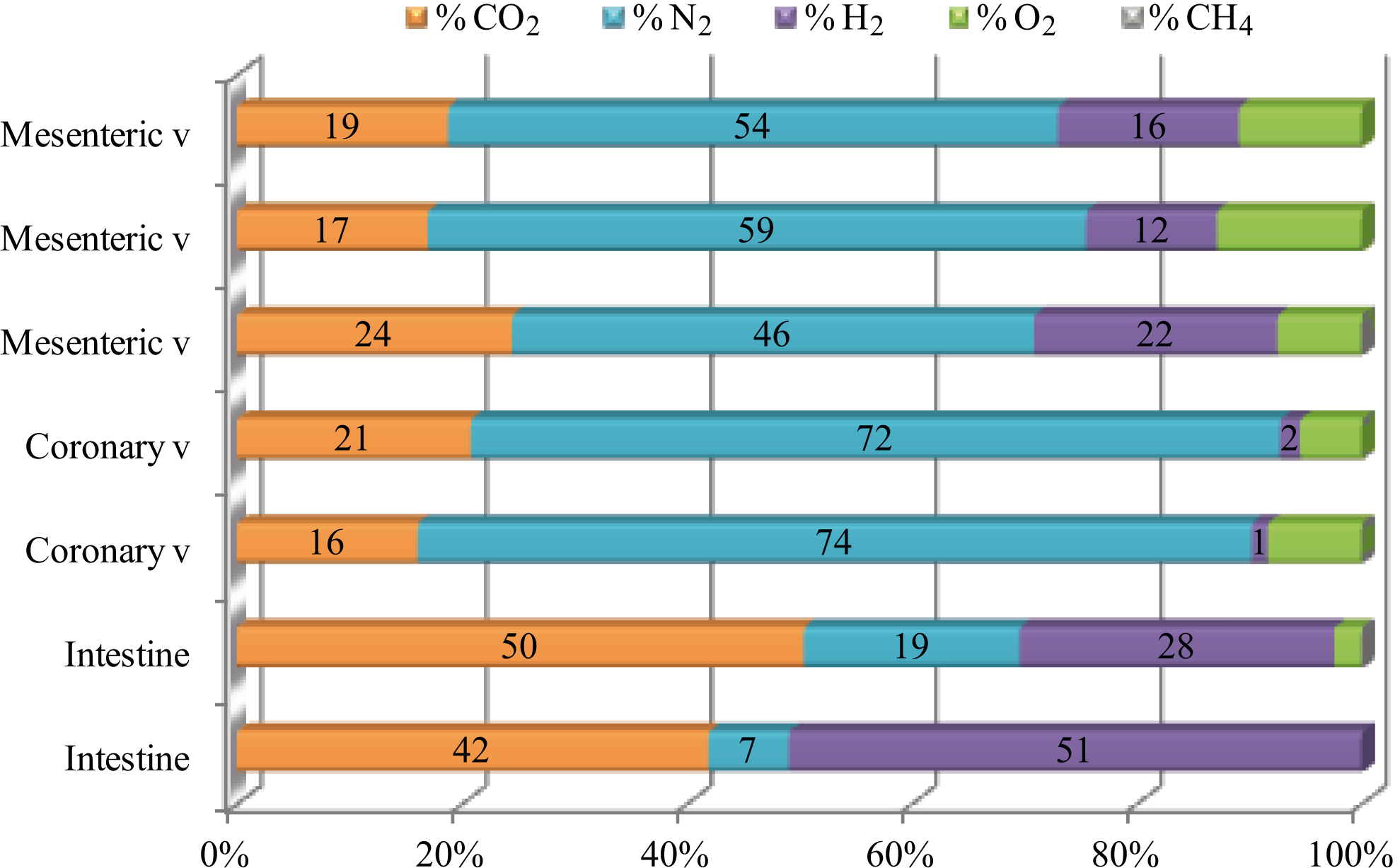

Supplement: Figure S8 — Gas composition of the bubbles found in D00193. Relative gas composition (% μmol) of samples taken at different body locations of D00193, a bycaught short beaked common dolphin. Abbreviations: v, vein. (TIF) [file pone.0083994.s008.tif]
